# Identifying the shared genes and KEGG pathways of Resolvin D1-targeted network and osteoarthritis using bioinformatics

**DOI:** 10.1080/21655979.2022.2061288

**Published:** 2022-04-18

**Authors:** Wenjun Jiang, Xiaoying Wang, Siwei Su, Sen Du, Hongqiang Song

**Affiliations:** Department of Sports Medicine and Rehabilitation, Shandong First Medical University (Shandong Academy of Medical Sciences), Taian, Shandong, China

**Keywords:** Bioinformatics analysis, chondrocytes, Resolvin D1, KEGG pathways, osteoarthritis

## Abstract

Osteoarthritis (OA) is a common chronic degenerative disease characterized by the loss of articular cartilage, which causes loss of joint function and reduce quality of life. Resolvin D1 (RvD1) has shown interesting anti-inflammatory effects; however, the mechanism of action of RvD1 in OA remains unclear. The aim of this study was to investigate the potential mechanism of RvD1 in OA by bioinformatics and partial in vitro mechanisms. Here, 106 shared differentially expressed genes (DEGs) were identified based on the GSE82107, GSE55235, GSE55457 dataset; 700 DEGs were identified based on GSE169077. Enrichment analyses of these genes were then successively conducted. RvD1-targeted genes and KEGG pathways are identified by STITCH. 27 shared KEGG pathways were identified among RvD1-targeted pathways and OA. Furthermore, cell apoptosis assay, western blotting, real-time fluorescent quantitative PCR (qRT-PCR), enzyme linked immunosorbent assay (ELISA) were used to confirm the expression levels of the key genes of shared Kyoto Encyclopedia of Genes and Genomes (KEGG) pathways between RvD1-targeted and OA in IL-1β treated rat knee chondrocytes. The results showed that RvD1-targeted pathways and the expression of nuclear p65, p53, and p-JNK were inhibited in the RvD1 group compared with the IL-1β group. Thus, the findings indicate that RvD1 may inhibit the development of OA through NF/kB, p53, MAPK/JNK, PI3K-AKT signaling pathways, and act as a treatment for OA.

## Graphical abstract

**Figure:**
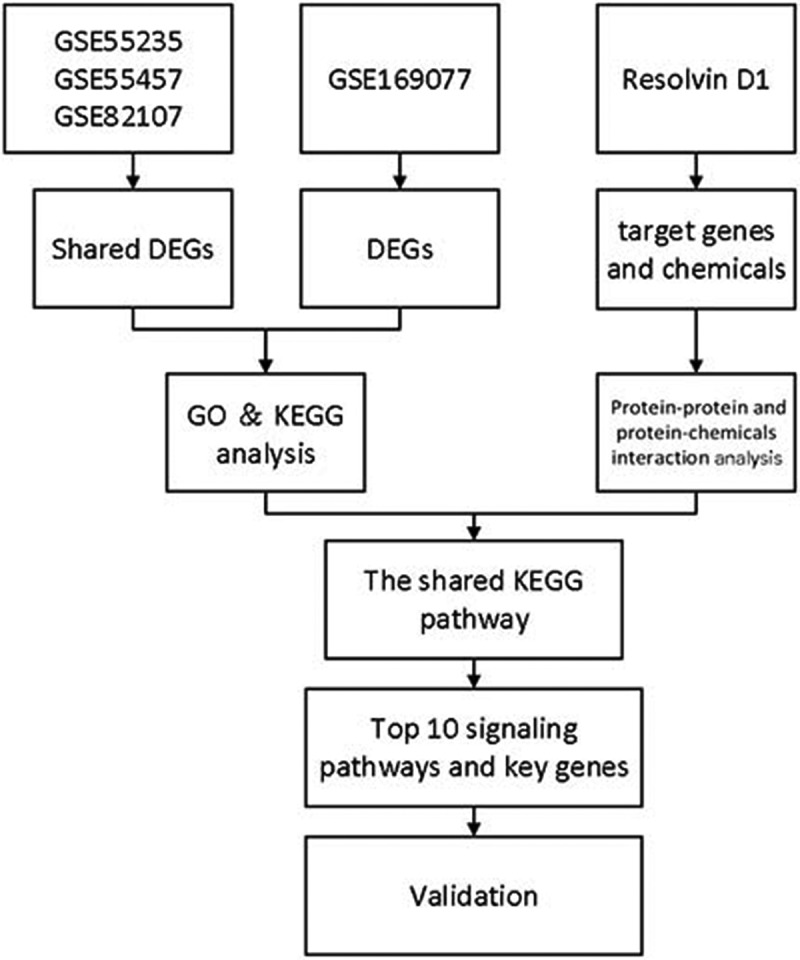

## Introduction

Osteoarthritis (OA) is a chronic, degenerative joint disease, which is mainly characterized by degeneration and destruction of articular chondrocytes, as well as bone, subchondral bone, and synovial membrane. This leads to joint deformity and dysfunction, seriously affecting the quality of life of middle-aged and elderly people [1,2]. Studies have shown that OA affects millions of people worldwide and places a huge economic burden on society [3]. However, the pathogenic mechanisms of OA are not clearly understood. There are no definitive drugs for osteoarthritis [4], most therapies by alleviating symptoms rather than curing OA in nature [5].

Resolvins are lipid derivatives generated from omega-3 polyunsaturated fatty acids, more specifically, after sequential enzymatic transformations of eicosapentaenoic acid and docosahexaenoic acid producing a series of E resolvins (RvE) and D resolvins [6]. Resolvin D1 (RvD1) has shown interesting anti-inflammatory effects and has been effective in the resolution of both acute and chronic inflammation [7,8]. It has also been reported that RvD1 has antioxidant and anti-fibrosis effects [9,10]. Benabdoune et al. [11] demonstrated that RvD1 can modulate inflammation, catabolism, and oxidative stress in IL-1β-induced chondrocytes, which may be a promising approach for OA treatment.

The Gene Expression Omnibus (GEO) database stores gene profiles containing microarray and sequence-based functional genomics [12,13]. To further clarify the mechanism of RvD1 in OA, bioinformatics analysis was used to identify the potential genes and signaling pathways between RvD1 and osteoarthritis, and provide evidence for the treatment effect of RvD1 in OA. Raw data from microarray analyses of GEO database were obtained from OA synovial membrane, OA cartilage tissue, and healthy controls. Previous studies has used the bioinformatic analysis of microarray datasets to identify the key genes in chondrocytes in OA [14], or used bioinformatic analysis to explore the biological markers of disease [15,16]. In this study, we explored the shared signaling pathways between RvD1-targeted genes and OA by Gene Ontology (GO) and Kyoto Encyclopedia of Genes and Genomes (KEGG) pathway functional enrichment analysis, protein-protein and protein-chemical interaction network analysis. The results and hub genes were validated using a cell apoptosis assay, western blotting, enzyme-linked immunosorbent assay (ELISA), and quantitative real-time PCR (qRT-PCR).

## Materials and methods

### OA-related data search and analysis

GEO database (www.ncbi.nlm.nih.gov/geo/) was searched to download OA-related gene expression and data for synovial membrane using GSE82107, GSE55235, GSE55457 datasets, thirty OA patients and twenty-seven normal controls were downloaded using GSE169077 dataset. The R software (version 3.6.3; https://www.r-project.org/) and limma package [17] were used to analyze the raw data. Differentially expressed genes (DEGs) were selected with p < 0.05, and |log2 fold change (FC)| >0.5 [18].

### RvD1-targeted genes search and prediction

The RvD1-targeted genes were searched using STITCH (http://stitch.embl.de/), which is a web‐based tool, that can identify and predict interactions between proteins, chemicals, and their related genes [19]. By importing ‘RvD1’ into STITCH, the interaction between RvD1 and its targets were calculated to generate an RvD1-targeted network. This was done using the following setting: medium confidence score 0.4; max number of interactors to show on shell: no more than 10 interactors.

### Functional and pathway enrichment analysis

The Database for Annotation Visualization and Integrated Discovery (DAVID 6.8) (http://david.abcc.ncifcrf.gov/) was used to analyze the Gene Ontology (GO) and Kyoto Encyclopedia of Genes and Genomes (KEGG) using R ‘ggplot2’ packages with statistically significant results (p < 0.05) [20].

### Gene set enrichment and KEGG pathway analysis

The protein-protein and protein-chemical interactions for RvD1 were determined using the STITCH (http://stitch.embl.de/) search function [19]. Cytoscape software (https://cytoscape.org/index.html) was used to reconstruct and visualize the network [21,22] of RvD1-targeted genes and chemicals. Then, the RvD1-targeted genes and related KEGG pathways also were analyzed.

### Exploring the KEGG pathways involved in both RvD1-targeted genes and OA

Gene ontology analysis was used to retrieve human OA KEGG pathways. Venn diagram (Venny 2.1, http://bioinfogp.cnb.csic. es/tools/venny/index.html) [23] was used to screen and visualize RvD1 targeted KEGG pathways using STITCH. The OA pathways were observed using the GEO database. The pathways were then selected using RvD1‐targeted genes (p < 0.05) that were also associated with OA.

### Cell isolation and culture

To further verify the effects of RvD1 on OA, rat knee joint chondrocytes were isolated and cultured. 15 male Sprague Dawley (SD) rats (7–10 days old) were purchased from the Experimental Animal Centre of Jinan, Shandong province, China. All animals were housed in the animal room of the Sports Medicine Institute of Shandong First Medical University. This study was approved by the Research Ethics Committee of Shandong First Medical University (Shandong Academy of Medical Sciences) (Grant No. 202,004,066). First, cartilage tissue was obtained under aseptic conditions from rats and dissolved in Dulbecco’s modified eagle Medium: Nutrient Mixture F-12 (DMEM/F12) containing 0.25% collagenase II (Solaibio, Beijing, China) at 37°C for 2 h. Chondrocytes were extracted and cultured in DMEM/F12 supplemented with 10% fetal bovine serum (biological industries, Kibbutz Beit-Haemek, Israel), 100 units/mL penicillin and 100 µg/mL streptomycin (biological industries, Kibbutz Beit-Haemek, Israel). The cells were cultured at 37°C and 5% CO_2_ in fresh medium until they coalesced and were ready for this experiment. All experiments were conducted using third passage cells. To identify chondrocytes by cell morphology, toluidine blue staining was used for glycosaminoglycans (Solaibio, Beijing, China) of the cultured cells.

### Cell treatments

The chondrocytes were treated with DMEM/F12 or 100 nM RvD1 (Cayman Chemical Company, Michigan, USA), 10 nM NF-κB inhibitor pyrrolidine dithiocarbamate (PDTC) (Aladdin, Shanghai, China) or the 10 nM JNK inhibitor SP600125 (Abcam, Cambridge, UK) or 10 μM p53 inhibitor PFTα (TargetMol, Boston, Massachusetts, USA) before specific tests. In addition to the control group, chondrocytes were seeded in culture plates and activated with DMEM/F12 containing 10 ng/mL IL-1β (Solaibio, Beijing, China) for 24 h to stimulate OA [24]. The cells were divided into the following groups: (1) Control group: cells were cultured with DMEM/F12. (2) IL‐1β group: cells were treated with DMEM/F12 containing 10 ng/ml IL‐1β for 24 h. (3) RvD1 group: cells were treated with 100 nM RvD1 for 3 h before being treated with IL‐1β. (4) PDTC group: cells were treated with 10 nM PDTC for 3 h before being treated with IL‐1β. (5) SP600125 group: cells were treated with 10 nM SP600125 for 3 h before being treated with IL‐1β. (6) PFTα group: cells were treated with 10 μM p53 inhibitor Pifithrin-α (PFTα) for 3 h before being treated with IL‐1β.

### RNA extraction and qRT-PCR

Total RNA was isolated from the cells using a simply P Total RNA Extraction Kit (Bioflux, Beijing, China) according to the manufacturer’s instruction, Total RNA (1 μg) was reverse transcribed to cDNA with starscript II First-stand cDNA synthesis Mix with gDNA Remover (GenStar, Winnipeg, Canada) as detailed in the manufacturer’s instructions. The amplification products were then detected using StepOne-Plus system (ABI, New York, USA). The primer sequences of genes were as follows: Interleukin-8 (*IL-8*) forward, 5′-TCCCGAATTGGAAAGGGAAATA-3′; reverse, CTGTTGGCCCAATTACTAACAG; tumor necrosis factor-α (*TNF-α*), forward, 5′-GCGCCCAGCCTTCCTTAC-3′, reverse, 5′-GCCCCGGCCT TCCAAATAAATAC-3′; glyceraldehyde-3-phosphate dehydrogenase (*GAPDH*), forward, 5′-GAAGGTCGGTGTGAACGGATTTG-3′, reverse, 5′-CATGTAGACCAT‐3′.

The relative expression level of mRNA of each gene was calculated using the 2ΔΔCt method and normalized to the internal reference GAPDH.

### ELISA

The supernatant of cultured cells was collected, and ELISA kits were used to test TNF-α (GenStar, Winnipeg, Canada) and IL-8 (mlbio, Shanghai, China), following the manufacturer’s instructions.

### Protein detection using western blotting

The cytoplasmic and nuclear proteins were extracted using the nucleoprotein and cytoplasmic protein extraction kit (KeyGEN Biotech, Jiangsu, China). The nuclear protein was used to detect the expression of p65, p53, Bax, p-JNK and JNK, and the total protein was extracted using highly efficient RIPA tissue/cell rapid lysate (Solaibio, Beijing, China) to test the expression of p65, p53, Bax, p-JNK and JNK and actin. Protein samples (30 µg) were loaded on a 12% sodium dodecyl sulfate-polyacrylamide gel for electrophoresis and then transferred to a polyvinylidene fluoride membrane. Subsequently, the membrane was blocked with blocking buffer for 1 h, then incubated with the following primary antibodies: p65 (CST, Boston, USA), p53 (CST, Boston, USA), Bax (CST, Boston, USA), p-JNK (CST, Boston, USA), JNK (CST, Boston, USA), actin (Santa Cruz, Dallas, USA), and proliferating cell nuclear antigen (Affinity Biosciences, Ohio. USA) at 4°C overnight. It was then incubated with secondary antibodies for 1 h.

### Cell apoptosis assay

To measure chondrocyte apoptosis, the Annexin V-FITC/propidium iodide (PI) apoptosis detection kit (meilunbio, Shanghai, China) was used for flow cytometry. The steps are outlined as follow: after treatment, chondrocytes were collected and immediately washed twice with ice-cold PBS, resuspended by 100 µL 1× Binding Buffer, and incubated with 5 µL Annexin V‐FITC and 5 µL PI for 10 min in dark at 26°C. Next, 400 µL 1× Binding Buffer was added. Finally, the total cell suspension was analyzed using flow cytometry. The results were calculated as the percentage of apoptotic cells (Annexin V+ PI−).

### Statistical analysis

All experiments were repeated at least thrice. Data were represented as means ± standard deviation; the SPSS 22.0 statistical software (IBM Corp., Armonk, New York, USA) was used to determine significant differences between the groups. Multiple sample means were compared using analysis of variance, and pair comparisons among multiple groups was performed using the Student–Newman–Keuls test. Statistical significance was set at p < 0.05.

## Results

To further explore the potential effect of Resolvin D1 in OA, bioinformatics analysis was used to identify the RvD1 targeted genes and signaling pathways that highly related to OA. Differentially expressed genes and enrichment analysis, the shared KEGG pathways of RvD1-targeted genes and OA analysis were performed. The biological effects of RvD1 and selected hub genes were verified by cell apoptosis assay, western blotting, qRT-PCR, ELISA. The aim of this study is to help us better understand the therapeutic effect of RvD1 in OA.

### Differential expression genes between OA and normal tissues

**Figure 1.**
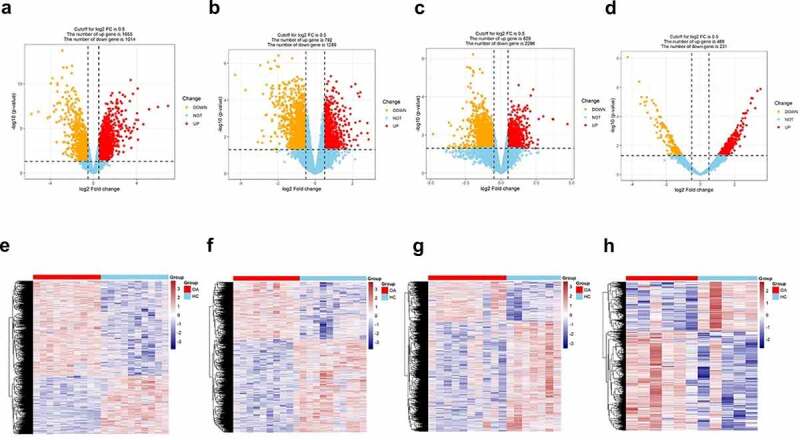
Differentially expressed genes screened between synovial tissue in osteoarthritis (OA) and normal control from GSE82107 (a, e), GSE55235 (b, f), GSE55457 (c, g) and GSE169077 (d, h) using volcano plot and heatmap. (a) 829 were upregulated and 2269 were down-regulated in GSE82107, (b)1655 were upregulated and 1014 were down-regulated in GSE55235, (c) 792 were upregulated and 1289 were down-regulated in GSE55457,(d) 469 were upregulated and 231 were down-regulated in GSE169077.The cutoff criteria were p < 0.05 and |log2 fold change (FC)| >0.5. Yellow, red, and blue dots represent downregulated, upregulated, and unchanged genes, respectively.

**Figure 2.**
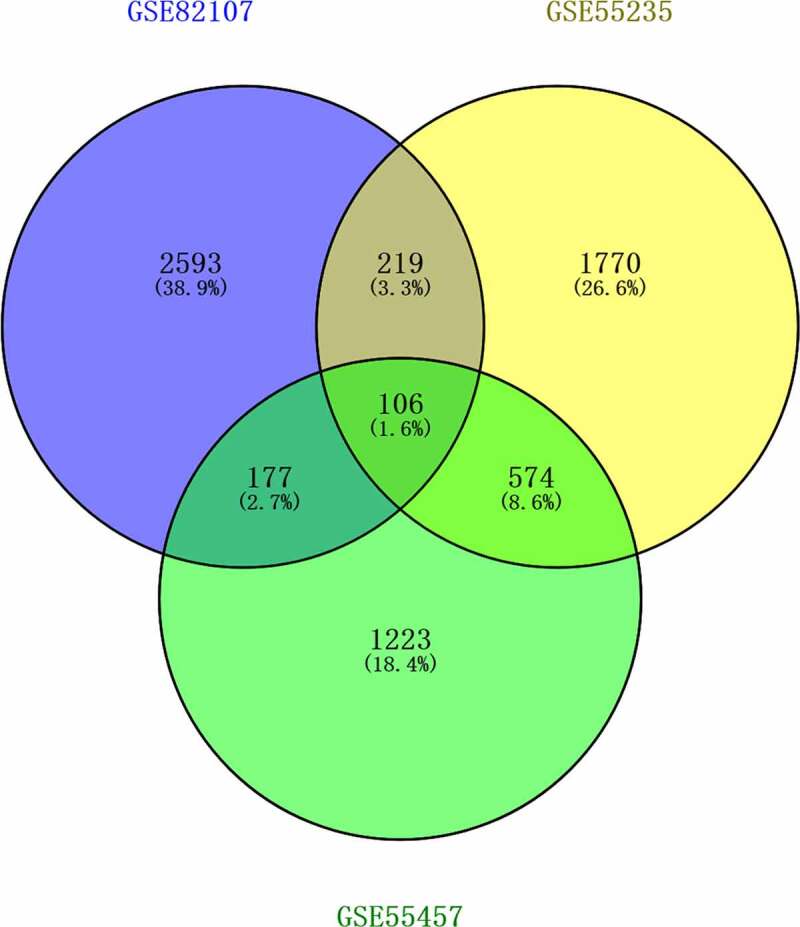
Venn diagram of shared differentially expressed genes screened between synovial tissue in OA and normal control from GSE82107, GSE55235, GSE55457. 106 shared differentially expressed genes from GSE82107, GSE55235, GSE55457. Blue circle represents GSE82107, yellow circle represents GSE55235, and green circle represents GSE55457.

**Figure 3.**
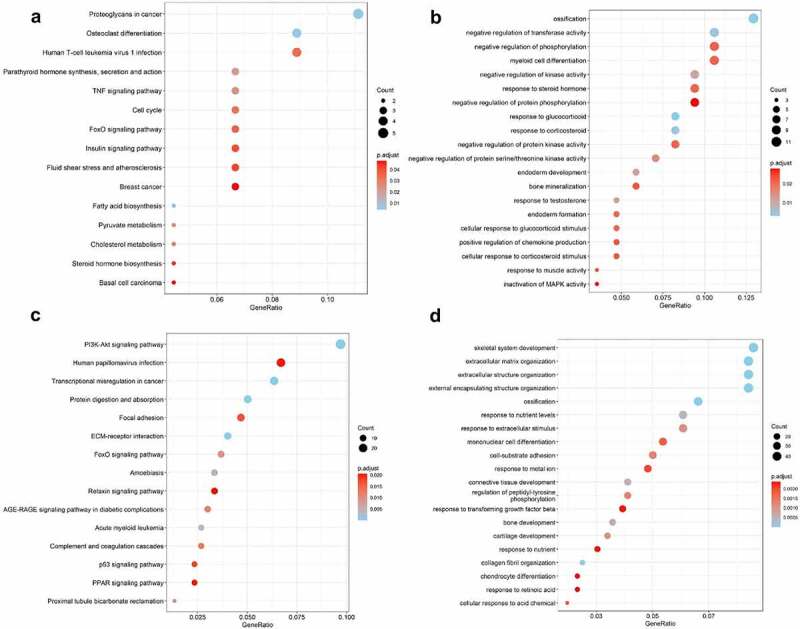
Bubble plots of Kyoto Encyclopedia of Genes and Genomes (KEGG) enrichment and Gene Ontology (GO) analysis of differentially expressed genes (DEGs). The KEGG enrichment and GO analysis of the shared DEGs from GSE82107, GSE55235, GSE55457 were shown in a, b. The KEGG enrichment and GO analysis of DEGs from GSE169077 were shown in c, d. The color indicates the p-value (from the lowest in blue to the highest in red), and the bubble size indicates the number of genes. The rich factor represents the proportion of the total number of genes.

The expression of genes was determined by processing the data from GSE82107, GSE55235, GSE55457 and GSE169077 were analyzed. The results were significant at the level of p < 0. 05 and |log2 fold change (FC)| >0.5. The level of differentially expressed genes (DEGs) is depicted by volcano plot, 829 were upregulated and 2269 were down-regulated in GSE82107 (Figure 1(a)), 1655 were upregulated and 1014 were down-regulated in GSE55235 (Figure 1(b)), 792 were upregulated and 1289 were down-regulated in GSE55457 (Figure 1(c)), 469 were upregulated and 231 were down-regulated in GSE169077 (Figure 1(d)); the DEGs in GSE82107, GSE55235, GSE55457 and GSE169077 were visualized by heat maps, respectively (Figure 1(e-h)). The shared DEGs of GSE55235, GSE55457, GSE82107 were showed by Venn diagram (Figure 2), 106 shared differentially expressed genes from GSE82107, GSE55235, GSE55457 were identified. The KEGG enrichment and GO analysis of the shared DEGs from GSE82107, GSE55235, GSE55457 were shown in Figure 3(a, b), the KEGG enrichment and GO analysis of the DEGs from GSE169077 were shown in Figure 3(c,d).

### RvD1-targeted genes and RvD1-linked networks

**Figure 4.**
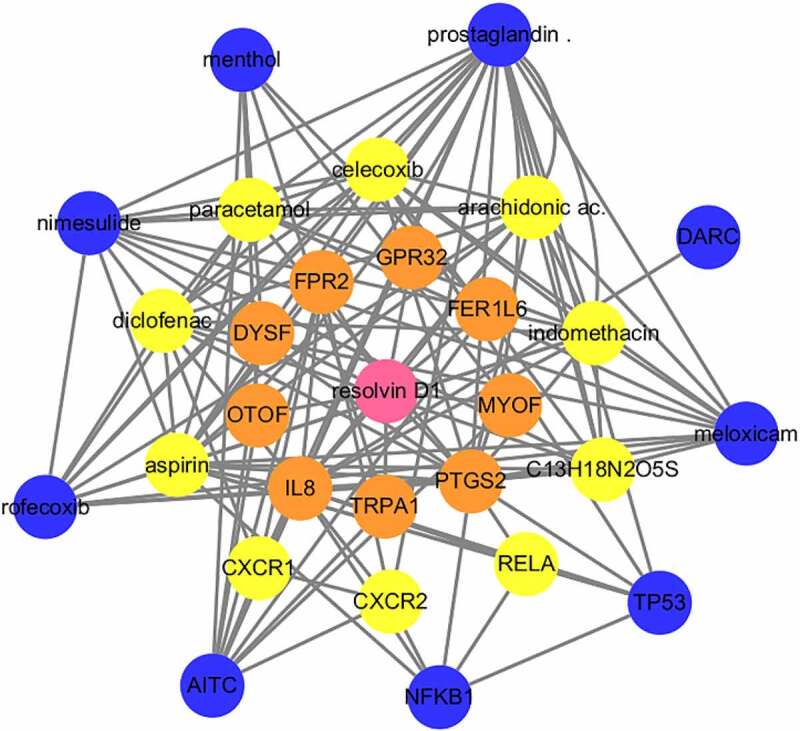
Visualization of the interaction networks of Resolvin D1 (RvD1)-targeted genes and chemicals constructed using Cytoscape. RvD1 targeted genes including: *IL8, TRPA1, PTGS2, MYOF, FER1L6, GPR32, FPR2, DYSF, OTOF, RELA, CXR2, CXR1, TP53, NFKB1, AITC, DARC*. The Orange circles represent the level of protein-protein interactions with RvD1. The luminous yellow circles and the blue circles represent the protein‐chemical interactions. Edges indicate interactions between circles.

To search for RvD1-targeted genes, STITCH (http://stitch.embl.de/) was used to input the Resolvin D1 and the search was conducted for *Homo sapiens*. STITCH is a database that integrates various chemicals. This process highlighted nine relevant genes in the first shell. The genes or chemicals were then expanded to the second and third levels. Sixteen target genes and twelve chemicals were thus identified. RvD1 targeted genes including: *IL8, TRPA1, PTGS2, MYOF, FER1L6, GPR32, FPR2, DYSF, OTOF, RELA, CXR2, CXR1, TP53, NFKB1, AITC, DARC*. The interactions of protein-protein and protein-chemical were constructed using Cytoscape (Figure 4).

### Shared KEGG pathways in both RvD1-targeted genes and OA

**Figure 5.**
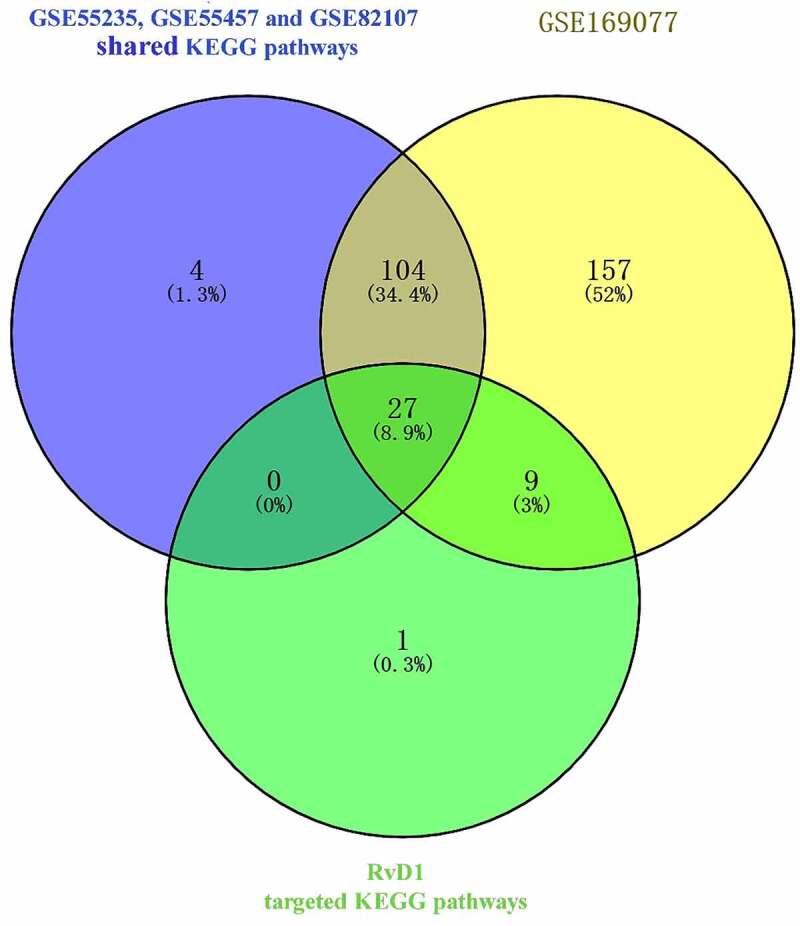
Venn diagram of identical shared KEGG pathways of RvD1‐targeted genes and OA related genes. Here, 37 RvD1‐targeted genes were related to KEGG pathways, 135 were human OA‐related KEGG pathways from synovial tissue and 297 were human OA‐related KEGG pathways from cartilage. There were 27 (8.9%) shared KEGG pathways.

Venn diagram was used to identified the shared KEGG pathways. A total of 37 related KEGG pathways of RvD1-targeted genes were identified; 135 human OA‐related KEGG pathways were retrieved from synovial tissue, 297 human OA‐related KEGG pathways were retrieved from cartilage by using the DAVID database. There were 27 shared KEGG pathways of RvD1-targeted genes and OA related genes as depicted with Venn diagrams (Figure 5). The top five shared pathways were small cell lung cancer, chemokine signaling pathway, apoptosis, prostate cancer, and the NF-kappa B signaling pathway. These pathways are involved many signaling pathways, to better understand the effect of RvD1 in OA via these pathways, the KEGG pathway database (https://www.genome.jp/kegg/pathway.html) [25] was used to analyze these top KEGG pathways. We found that they mainly include nuclear factor-κB (NF-κ B), p53, MAPK, PI3K-Akt signaling pathways and others. The top ten shared pathways are displayed in Table 1.

**Table 1.** The top four shared Kyoto Encyclopedia of Genes and Genomes (KEGG) pathways of differentially expressed genes of osteoarthritis and RvD1 targeted genes

ID	KEGG pathway	RvD1-target genes	p value
hsa05222	Small cell lung cancer	PTGS2, TP53, NFKB1, RELA	7.09E-05
hsa04062	Chemokine signaling pathway	CXCR1, CXCR2, NFKB1, RELA	5.19E-04
hsa04210	Apoptosis	TP53, NFKB1, RELA	1.87E-03
hsa05215	Prostate cancer	TP53, NFKB1, RELA	3.27E-03
hsa04064	NF-kappa B signaling pathway	PTGS2, NFKB1, RELA	3.34E-03
hsa04668	TNF signaling pathway	PTGS2, NFKB1, RELA	4.69E-03
hsa05200	Pathways in cancer	PTGS2, TP53, NFKB1, RELA	5.26E-03
hsa05169	Epstein-Barr virus infection	TP53, NFKB1, RELA	5.31E-03
hsa05160	Hepatitis C	TP53, NFKB1, RELA	7.18E-03
hsa05161	Hepatitis B	TP53, NFKB1, RELA	9.06E-03

### Identification of cultured chondrocytes

**Figure 6.**
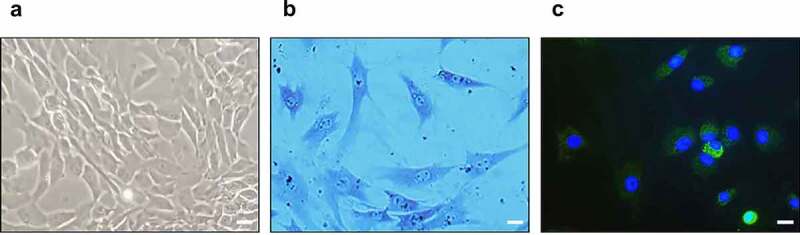
Identification of cultured chondrocytes. (a) Optical microscopy of cultured chondrocytes (400x). (b) Toluidine blue staining of cultured chondrocytes (400x). (c) Immunofluorescence staining for type-II collagen (green) and 4′,6-diamidino-2-phenylindole (blue) (400x), the scale bar is 20 μm.

To identify the cultured chondrocytes, toluidine blue staining and immunofluorescence staining for type-II collagen were performed. In the optical microscopy results of the cultured chondrocytes, most chondrocytes were observed to be spherical (Figure 6(a)). In addition, toluidine blue staining for glycosaminoglycan (Figure 6(b)) and immunofluorescence staining for type-II collagen and 4′,6-diamidino-2-phenylindole were positive (Figure 6(c)).

### RvD1 inhibits IL‐1β‐induced chondrocytes apoptosis

**Figure 7.**
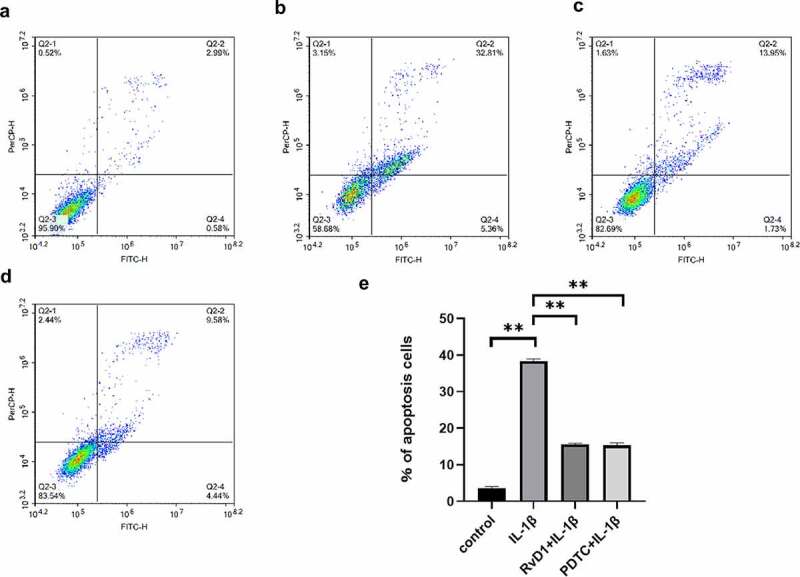
The results of Annexin v- FITC /PI. (a) Chondrocyte apoptosis following DMEM/F12 treatment. (b) Chondrocyte apoptosis following IL‐1β treatment. (c) Chondrocyte apoptosis following RvD1+ IL-1β treatment. (d) Chondrocyte apoptosis following PDTC+IL‐1β treatment. (e) The analysis results for the percentage of apoptosis among the four groups. **p < 0. 01.

To verify the anti-apoptotic effect of RvD1 in OA, cell apoptosis was assessed using flow cytometry after the chondrocytes were treated with DMEM/F12 (Figure 7(a)), IL-1β (Figure 7(b)), RvD1+ IL‐1β (Figure 7(c)), or PDTC+IL‐1β (Figure 7(d)). The results suggested that the IL‐1β group had a higher rate of cell apoptosis than the other groups (p < 0.01); however, the cell apoptosis rate of the RvD1+ IL‐1β group was lower than that of the IL‐1β group (p < 0.01; Figure 7(e)).

### RvD1 inhibits production of proinflammatory cytokine in IL-1β-treated chondrocytes

**Figure 8.**
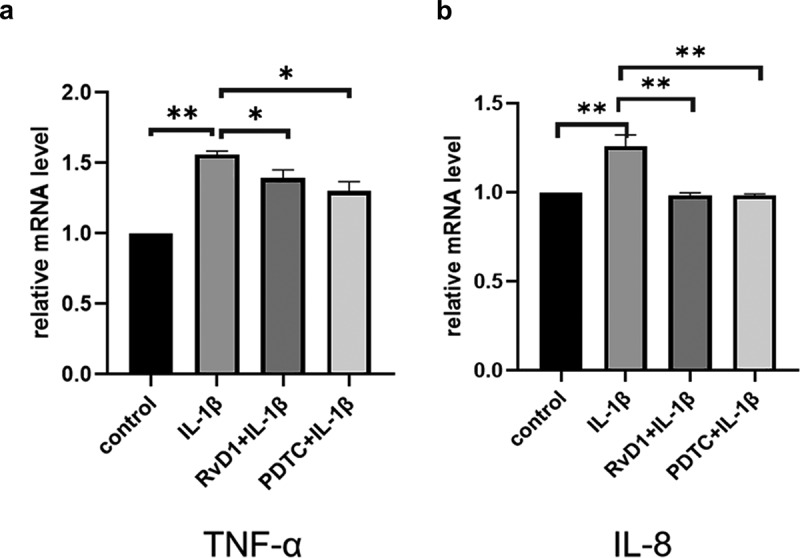
The result of qRT‐PCR of proinflammatory cytokines in IL-1β-treated chondrocytes. Cells were incubated with DMEM/F12, IL‐1β, RvD1+ IL-1β, and PDTC+IL‐1β; the total RNA was collected. RvD1 and PDTC inhibited the gene expression of (a) TNF-α and (b) IL‐8. All experiments were repeated three times. *p < 0.05, **p < 0. 01.

**Figure 9.**
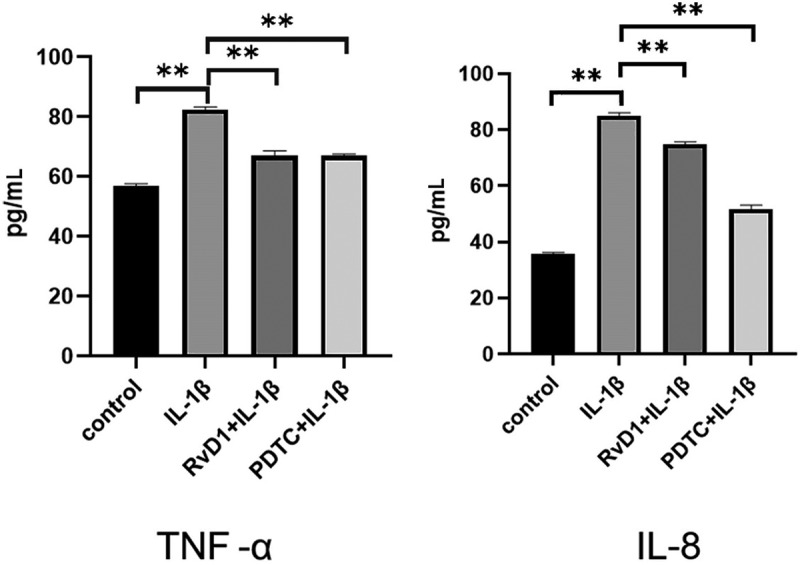
The result of enzyme-linked immunosorbent assay (ELISA) of proinflammatory cytokines in IL-1β-treated chondrocytes. Cells were incubated with DMEM/F12, IL‐1β, RvD1+ IL-1β, and PDTC+IL‐1β. The levels of (a) TNF-α and (b) IL‐8 were assessed with the ELISA kit. RvD1 and PDTC decreased the level of TNF-α and IL‐8. All experiments were repeated three times. **p < 0.01.

To evaluate the anti-inflammatory effect of RvD1 in OA according to the RvD1-targeted genes and signaling pathways, cells were incubated with DMEM/F12, IL‐1β, RvD1+ IL‐1β, or PDTC+IL‐1β; qRT-PCR was used to detect the expression of related genes by extracting total RNA (Figure 8(a,b)). In addition, the concentrations of TNF-α and IL-8 in the cell supernatant were detected using ELISA (Figure 9(a,b)). The results show that the IL‐1β group had a higher level of TNF-α and IL-8 than the other groups (p < 0.01), and RvD1 inhibited the expression of TNF-α (p < 0.01) and IL-8 (p < 0.01).

### RvD1 inhibits the activation of NF-κB signaling pathway

**Figure 10.**
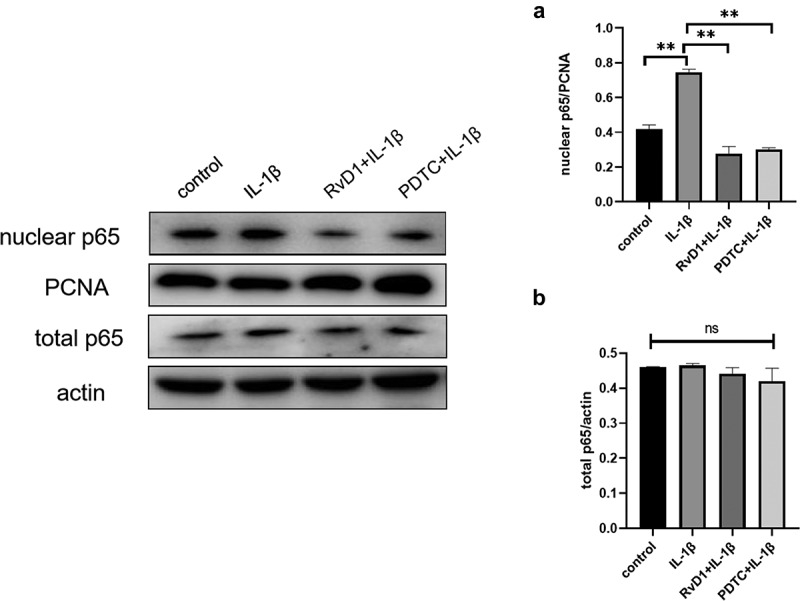
RvD1 inhibits the activation of NF‐κB signaling pathway in IL-1β induced chondrocytes. The results of western blotting of (a) nuclear p65 and (b) total p65. RvD1 and PDTC were shown to decrease the expression of nuclear p65. All experiments were repeated three times. **p < 0.01, ns indicates no significant difference.

According to the results of bioinformatics analysis, *TP53, NFKB1, RELA* are the key RvD1-targeted genes, NF-κB signaling pathway is one of top signaling pathway. To verify these results, the level of nuclear p65, total p65 were detected using western blotting. After treatment of chondrocytes with IL-1β, the expression level of nuclear p65 was higher than that in the control group (p < 0.01; Figure 10(a)). In addition, for the RvD1+ IL-1β and PDTC+IL-1β group, the effect of IL-1β on activating the NF-κB pathway was inhibited, the expression of nuclear p65 was also inhibited in the RvD1 and PDTC groups compared with the IL-1β group (p < 0.01; Figure 10(a)). These results indicate that RvD1 may inhibit NF-κB pathway through preventing p65 nuclear translation.

### RvD1 inhibits the p53 signaling pathway to suppress cell apoptosis

**Figure 11.**
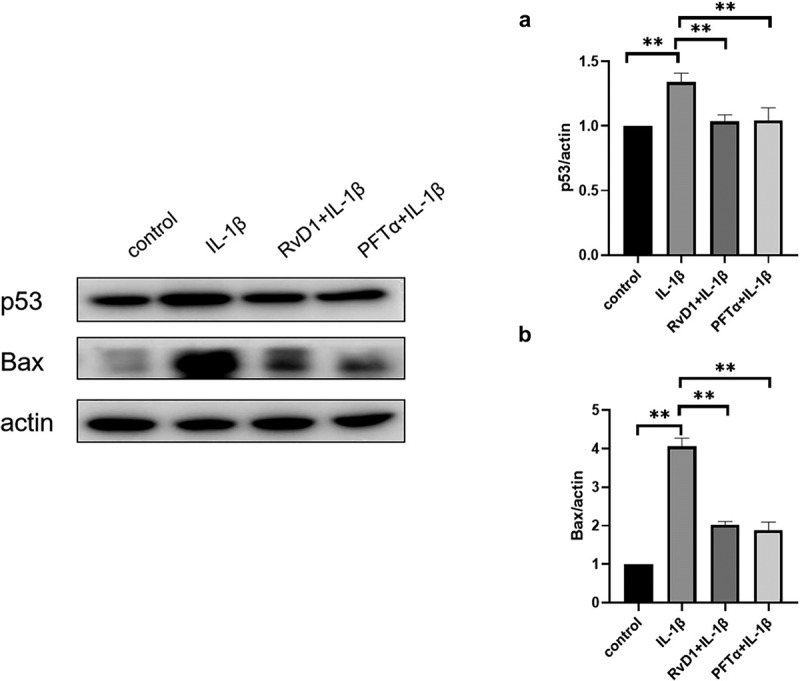
RvD1 inhibits chondrocytes apoptosis via p53 signaling pathway. The results of western blotting of (a) p53 and (b) Bax. RvD1 and PFTα were shown to decrease the expression of nuclear p53 and Bax. All experiments were repeated three times. *p < 0.05, **p < 0.01.

To further explore the anti-apoptotic effect of RvD1 on p53 signaling pathway, the expression of p53 and Bax was detected by western blotting. After treatment of chondrocytes with IL-1β, the expression level of p53 and Bax were higher than that in the control group (p < 0.01). The expressions of p53 (Figure 11a) and Bax (Figure 11b) were suppressed in the RvD1 group (p < 0.01). These results showed that RvD1 may inhibits the activation of p53 signaling pathway.

### RvD1 suppress the activation of MAPK/JNK signaling pathway

**Figure 12.**
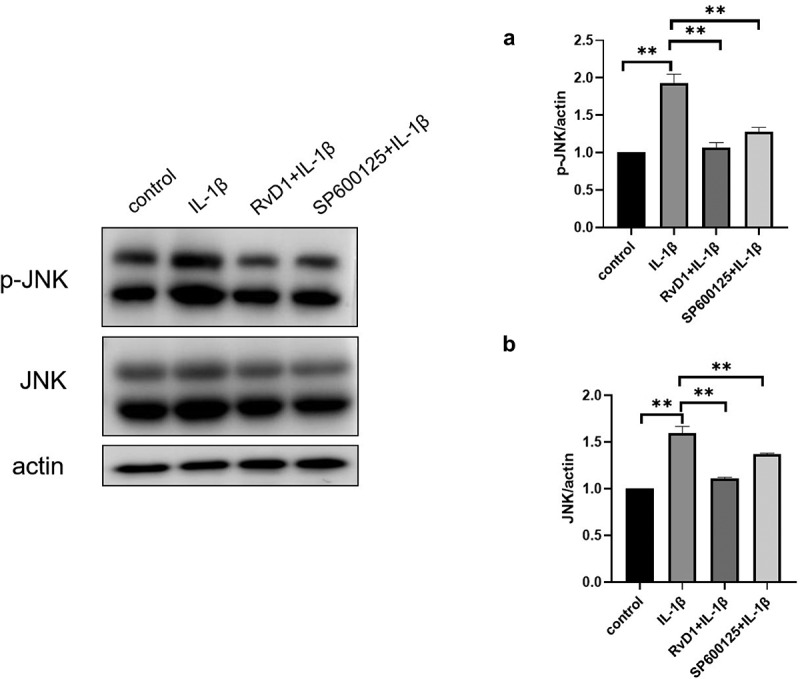
RvD1 inhibits the activation of MAPK/JNK signaling pathway. The result of western blotting of (a) p-JNK and (b) JNK. RvD1 and SP600125 were shown to decrease the expression of p-JNK. All experiments were repeated three times. **p < 0.01.

To further identify the mechanism of action of RvD1, the expression of JNK and p-JNK was analyzed using western blotting. As shown in Figure 12, the level of p-JNK was significantly increased in the IL‐1β group compared to the control group. However, in the RvD1 group, the level of p-JNK was significantly lower than that in the IL‐1β group (Figure 12(a)).

## Discussion

OA is a degenerative bone and joint disease that usually occurs in middle-aged and elderly individuals [2,26]. Studies have proved that older age, trauma, and obesity are associated with OA [27–30], with increasing research focused on finding treatments for the symptoms and preventing OA progression.

In this study, synovial tissue and cartilage samples from patients with OA were analyzed using GSE82107, GSE55235, GSE55457, and GSE169077. RvD1-targeted genes and KEGG signaling pathways were identified by STITCH. In comparision with previous studies, we analyzed the differential gene expression of synovial tissue and cartilage in OA as well as potential shared signaling pathways among synovial tissue, cartilage in OA, and RvD1-targeted genes. Additional experiments were conducted to verify these results which could help to understand the pathological mechanism of OA and provide data supporting the use of RvD1 in the treatment of OA.

The results show that there are 37 potential KEGG pathways of RvD1-targeted genes. We found 131 shared KEGG pathways between synovial tissue and cartilage sample in OA, and 27 shared KEGG pathways among synovial tissue, cartilage sample in OA, and RvD1-targeted pathways (Figure 5). The top five shared KEGG pathways are small cell lung cancer, chemokine signaling pathway, apoptosis, prostate cancer, NF-kappa B signaling pathway. The hub genes include *TP53, NFKB1, RELA* (Table 1). In addition, RvD1 targets *IL-8* (Figure 4). The KEGG pathway of small cell lung cancer, chemokine signaling pathway, apoptosis, and prostate cancer involve many signaling pathways, to better understand the effect of RvD1 in OA via these pathways, the KEGG pathway database (https://www.genome.jp/kegg/pathway.html) was used to analyze these top KEGG pathways. We found that they include nuclear factor-κB (NF-κ B), p53, MAPK, PI3K-Akt signaling pathways and others. Therefore, we supposed that RvD1 exerts biological effects mainly through NF-κB, p53, MAPK, PI3K-Akt signaling pathways. These pathways are involved inflammation, apoptosis, glucose transport, and cell signal transduction. The results validated using qRT-PCR and ELISA showed that the mRNA levels and expressions of TNF-α and IL-8 (Figure 8 and 9) were significantly decreased in the RvD1 group compared to the IL-1β group. In addition, western blotting was used to detect the expression of nuclear p65, p53, and Bax, JNK, and p-JNK, and the results showed that RvD1 significantly inhibit the expression of these proteins.

NFKB1 (p105/p50) and RELA (p65) are the numbers of the Rel/NFkB family, and the p65 (RelA) plays a key role in the classical NF-κB signaling pathway [31–33], NF-κB regulates apoptosis, inflammation, and immune response. Previous studies revealed that NF-κB regulates the development of OA, and inflammatory activities of TNF-α,IL-8 and others. In OA chondrocytes, TNF-α, IL-8, and other catabolic cytokines are generated, increasing the secretion of metalloproteinases [34], reducing the production of proteoglycan and collagen, and promoting articular damage [35]. In this study, the results showed that RvD1 inhibited the expression of nuclear p65 (Figure 10) and decreased the levels of TNF-α and IL-8 in IL-1β-induced chondrocytes. Therefore, we supposed that RvD1 inhibits the NF-κB signaling pathway to suppress production of proinflammatory cytokine and cell apoptosis.

p53 and NF-κB are major important transcription factors that control cell death and survival [36]. p53 is an important tumor suppressor, and it has been well studied in cancer treatment because of its functions in apoptosis, metabolism, cell cycle arrest, autophagy, and DNA repair. The activation of p53 can induce the expression of Bax, Bcl2, and related apoptotic proteins [37–39]. Studies have shown that the expression level of p53 is higher in patients with knee OA than in healthy controls [40]; p53 plays an indispensable role in OA [41,42]. However, the mechanism of p53 action in arthritis remains unclear. In this study, the expression of p53 and Bax was inhibited in the RvD1 group, the results showed that RvD1 inhibit chondrocyte apoptosis through suppressing the activation of p53 signaling pathway in OA (Figure 11).

MAPK is a serine-threonine protein kinase family that includes p-JNK/JNK, p-p38/p38, and p-ERK/ERK. MAPK regulates cell differentiation, proliferation, inflammation, apoptosis, and innate immunity [43]. Previous studies have shown that MAPK plays a vital role in chondrogenic differentiation [44,45]. In this study, the expression level of p-JNK significantly decreased in the RvD1 group (Figure 12), it indicates that RvD1 may alleviate chondrocytes injury via inhibiting the MAPK /JNK pathway in OA.

The PI3K-Akt signaling pathway is important for the cell cycle and is an intracellular signaling pathway, that also plays a role in cell survival, inflammation, apoptosis, and metabolism [46]. When PI3K is activated, downstream effectors, such as AKT and mammalian target of rapamycin complex 1 (mTORC1) are activated [47]. In recent years, many studies have shown that the PI3K/AKT/mTOR signaling pathway plays an irreplaceable role in normal knee metabolism in OA [48]. A previous study showed that inhibition of the PI3K/AKT/mTOR signaling pathway can alleviate inflammation and promote rat chondrocyte autophagy in OA [49]. In this study, we found that PI3K-Akt signaling pathway is one of the key shared pathways between RvD1 targeted genes and OA. We supposed that RvD1 also has an effect on PI3K-Akt signaling pathway in OA.

Here, bioinformatics analysis was used to identify the shared hub genes and signaling pathways between RvD1-targeted network and OA, and the results were validated using qRT-PCR, ELISA, and western blotting; These results provide evidence for possible treatment of RvD1 in OA and help further understand the mechanism of OA. This study is limited by the fact that the hub genes and signaling pathways were verified using IL-1β-induced chondrocytes, and these results need to be validated in human OA chondrocytes.

## Conclusion

In this study, the shared genes and signaling pathways between targeted RvD1 and OA were identified with *TP53, NFKB1, and RELA* as the hub genes. Our results show that RvD1 may suppress the development of OA via the NF‐κB, p53, MAPK/JNK, and PI3K-Akt signaling pathways. These findings indicate that RvD1 may serve as a therapeutic agent for OA.

## Supplementary Material

Supplemental Material

## Data Availability

The datasets used or analysed during the current study are available from the corresponding author upon reasonable request.
